# Adjunct Hypertonic Saline in Patients with Diffuse Edema Due to Heart Failure: A Randomized Double-Blinded Clinical Trial

**DOI:** 10.22037/ijpr.2020.113853.14526

**Published:** 2021

**Authors:** Mohammad Parsa Mahjoob, Farnaz Barzi, Amirahmad Nassiri, Alireza Kaveh, Mahshid Haghi, Mahshad Ghoddusi, Mohammad Sistanizad

**Affiliations:** a *Cardiovascular Research Center, School of Medicine, Shahid Beheshti University of Medical Sciences, Tehran, Iran. *; b *Imam Hussein Hospital, Shahid Beheshti University of Medical Sciences, Tehran, Iran. *; c *Emam Hossein Medical Center, Shahid Beheshti University of Medical Sciences, Tehran, Iran. *; d *Shahid Moddares Hospital, Shahid Beheshti University of Medical Sciences, Tehran, Iran. *; e *Department of Medical Nanotechnology, Faculty of Advanced Sciences and Technology, Pharmaceutical Sciences Branch, Islamic Azad University, Tehran, Iran.*; f *Department of Clinical Pharmacy, Faculty of Pharmacy, Shahid Beheshti University of Medical Sciences, Tehran, Iran. *; g *Prevention of Cardiovascular Disease Research Center, Shahid Beheshti University of Medical Sciences, Tehran, Iran.*; h Haghi^e^, Mahshad Ghoddusi^c^ and Mohammad Sistanizad^f, g*^

**Keywords:** Hypertonic saline solution, Furosemide, Diuretic resistant, Generalized edema, Chronic heart failure

## Abstract

In patients with diuretic resistance due to heart failure, higher doses or continuous furosemide infusion and adding hypertonic saline solution (HSS) to diuretics could be effective. The goal of this study was to assess the effectiveness of hypertonic saline solution administration in weight loss of hospitalized patients with diuretic-resistant edema due to heart failure. In a randomized double-blinded clinical trial, adult patients with diffuse peripheral edema due to heart failure who were unresponsive to 80 mg of oral furosemide were enrolled. The patients were randomized into two groups. In the intervention and control groups, patients received 150 mL of HSS and normal saline, respectively. Subjects in both groups received 250 mg IV furosemide every 12 h for 48 h. The change in body weight, urine output, blood pressure, uric acid, urine osmolality, blood biochemistry, and urinary cystatin C levels were assessed. Based on defined inclusion and exclusion criteria, 28 patients, 14 in each group, were recruited. The groups were similar in demographic and baseline laboratory characteristics. A significant decrease in body weight was observed in the intervention group (*P* = 0.002). The change in other measured parameters, including urine output and urinary cystatin C levels, was not reached statistical significance. Our findings suggest that the administration of HSS as an adjunct to loop diuretics could provide a safe and effective treatment for increasing urine output and decreasing weight in patients with heart failure.

## Introduction

Edema of advanced diseases such as Chronic Heart Failure (CHF), nephrotic syndrome, and cirrhosis (1) is a common, multifactorial feature, which debilitates patients’ quality of life (2). A typical treatment with diuretics is commonly ineffective and could even be harmful (3, 4). Also, chronic treatment with these agents may result in a phenomenon called “chronic braking” which limits the diuretic response and deteriorates the clinical condition of the patients (5). Age-related impairment of renal function, adjuvant therapies that may affect renal function (6), changes in gastrointestinal function induced by CHF, and the reduced splanchnic blood flow (7) contribute to this resistance. 

In patients with diuretic resistance, the proposed therapeutic option includes intravenous boluses of 160–200 mg of furosemide. Other strategies include more frequent administration, continuous infusions, or the addition of a thiazide/metolazone to block distal nephron sodium (Na) reabsorption. Additionally, these subjects may be treated with a combination of furosemide and human albumin (7, 8). Several studies have determined the efficacy of Hypertonic Saline Solution (HSS) infusion in situations in which regional organ blood flow is impaired (7, 9 and 10). The proposed mechanisms could be direct myocardial stimulation with high cardiac output maintenance, an increase in intravascular volume and subsequent peripheral arterial vasodilatation, a reduction of tissue edema (shifting of tissue water along the osmotic gradient), increased renal blood flow, and reduced sympathetic tone (11). These findings have allowed for greater weight loss, a more significant reduction in neurohormonal activation, a shorter hospital stay time, and a significant recovery in renal function (12). 

This study aimed to investigate the effectiveness of HSS administration in weight loss of hospitalized patients with diuretic-resistant generalized edema due to heart failure as a primary objective. 

## Experimental

*Population*

This is a randomized, double-blinded clinical trial performed on patients admitted to the post CCU ward of Imam Hussein Hospital, a 600-bed hospital affiliated to Shahid Beheshti University of Medical Science (SBMU) in Tehran, Iran; from April 2018 to February 2019. The target population for this trial was patients older than 18 years old with diffuse peripheral edema who did not respond adequately to 80 mg oral furosemide, including patients with proteinuria and edema resistant to treatment and nephrotic syndrome). 

*Exclusion Criteria*

Patients with the following conditions were excluded from the study: candidates for hemodialysis, systolic blood pressure ≤80 mmHg, GFR ≤15 mL/min, serum albumin ≤2.5 g/dL, serum potassium ≥5.5 mEq/L, serum sodium greater than 145 mEq/L, and anuric patients (urine volume ≤100 mL per 24 h). Besides, patients with comorbidities, as dementia, cerebral vascular disease and inability to give informed consent were excluded, as well as patients requiring pacemaker implantation, those with a substance or alcohol addiction and patients who were receiving mineralocorticoids. 

*Study Protocol*

All participants were evaluated carefully based on the mentioned inclusion and exclusion criteria and eligible subjects were recruited after the completion of the written consent form. For all participants, demographic data, past medical and habitual history were documented. A cardiologist examined patients, and chest radiographs, electrocardiograms and echocardiograms for determination of ejection fraction were performed at the beginning of the study. 

Subjects were randomized using permuted block randomization into two groups. In the intervention group (A), patients received 150 ml of hypertonic saline solution (NaCl 5%) plus 250 mg of intravenous furosemide. In the control group (B), patients received 150 mL of normal saline plus 250 mg IV furosemide every 12 h for 48 h. For patients receiving other diuretics, including spironolactone and thiazides, the agent was continued with the previous dose. A new diuretic agent was not added to the treatment regimen during the intervention period. 

All patients were weighed before (baseline) and 72 h after the intervention (3^rd^ day of the study) by digital scale (Beurer, Germany). Besides, the amount of urine output (all patients had foley catheter), serum levels of Na, K, Cl, uric acid, creatinine (SCr), urea, urine random Na, urine osmolality, and urinary cystatin-C were determined at baseline and 3^rd^ day of the study. 

In order to prevent hypokalemia, as a complication of furosemide, both groups received intravenous potassium to maintain serum potassium at levels greater than 3.5 mEq/L.

In order to detect possible kidney injury due to the administration of large doses of furosemide, besides Scr, we also followed urinary cystatin-C levels. For measurement of urinary cystatin-C levels, 5 mL of urine was collected, centrifuged at 10,000g at 4 °C for 2 min, and then the supernatant was aliquoted and stored at -80 °C. Urinary cystatin-C level was measured using Human Cystatin C ELISA Kit (Abcam, United Kingdom) using the protocol mentioned in the kit’s brochure. 

*Statistics*

The data analysis was done by the statistical package for social sciences (SPSS) software version 21.0 (Chicago, IL, USA). Descriptive statistics were used for quantitative variables (mean ± standard deviation). The Kolmogorov-Smirnov test was used to determine whether the study population followed the normal distribution. The difference between the two treatment group data was analyzed using the Chi-square test, Mann-Whitney U test and Independent Sample T-test. A *P*-value of less than 0.05 was considered statistically significant.

*Sample size*

Considering the patient population based on inclusion and exclusion criteria, we considered 15 patients in each group.

*Ethics*

All patients signed informed consent and the benefits and complications were explained to them before entering the study. All stages of the study have been supervised by the medical ethics committee of Imam Hussein hospital of Shahid Beheshti University of Medical Sciences. This study has been approved by the ethical committee of Shahid Beheshti University of Medical Sciences (IR.SBMU.retech.rec.1397.144) and was registered in Iranian Registry of Clinical Trials (IRCT) platform with a code of IRCT20200812048380N1.

## Results

**Figure 1:**
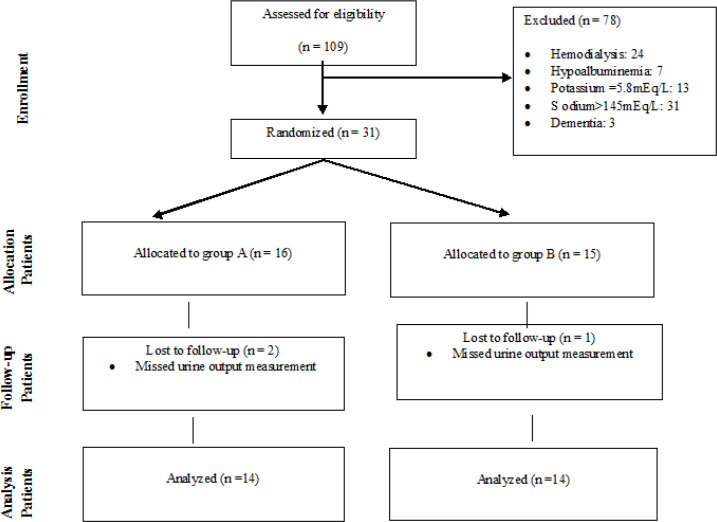
Consort chart of the study

Twenty-eight patients (13 women and 15 men) were evaluated based on defined inclusion and exclusion criteria. Figure 1 reveals the consort chart of the study.

The baseline demographics and clinical characteristics (drug history, past medical, and habitual history) did not significantly differ between the patients in the two arms of the study. At the baseline, there was no significant difference between the two groups in terms of weight, blood pressure, heart rate, urine osmolality, urine output, cystatin C and serum electrolyte, albumin, and creatinine (Table 1).

As shown in Table 2, on the third day of the study, there was no significant difference in measured parameters between the two arms of the study. 

Urinary cystatin C levels were 2.44 ± 0.90 *vs.* 2.04 ± 0.80 (*P* = 0.23) in the control and intervention arms of the study, respectively. We also analyzed the amount of changes in the two arms. The urine output increased 589 ± 1254 mL and 1028 ± 649 mL in the control and intervention arms of the study, respectively (*P* = 0.255). Patients weight decreased 3.59 ± 2.12 kg and 6.38 ± 2.17 kg which was statistically significant (*P* = 0.002). Changes in other parameters did not reach a significant level. 

*Power Analysis*

Based on the primary objective of weight change, the power for the mean of control group = mean of intervention group plus the difference, difference of 2.79, SD of 2.12, a sample size of 13 in each group and α = 0.05, the power calculated as 0.95. 

## Discussion

While refractory fluid overload remains a symptom affecting CHF, nephrotic syndrome, and cirrhosis patients (13), its effective management remains clinically challenging. A combination of HSS with loop diuretics may be a probable alternative in the treatment of refractory fluid retention. A review of the literature proved somewhat few investigations regarding the combination management of HSS with furosemide in palliative care management.

This study showed that despite the non-significant increase in urine output, patients with fluid retention and treated with a combination of high dose furosemide (250 mg twice daily), and HSS reached a greater weight loss than those treated with just high dose diuretic. 

In accordance with other studies (7, 14-16), these results demonstrate that the Na management in acute phases of subjects with fluid retention plays a critical role in maintaining euvolemic status and prevents plasma volume decline as it occurs using the diuretic treatment.

HSS infusion achieves a rapid ascent of extracellular NaCl concentration, with a subsequent rise in osmotic pressure, plasmatic volume expansion, instantaneous fluid mobilization into the vascular compartment, and increased renal blood flow (7). Furthermore, fluid shifted out of erythrocytes and endothelial cells to the extracellular space causes a reduction in capillary hydraulic resistance (14). The accelerated increase of extracellular fluid volume is responsible for the diminished plasma and peritubular oncotic pressure that, with an increased peritubular hydrostatic pressure, enhances the urinary Na excretion by a decline in proximal Na reabsorption (15). Actually, due to its osmotic effect, HSS causes a rapid and spontaneous mobilization of fluids from the third space to the vascular compartment without a significant simultaneous rising of serum Na (12). The concurrent administration of furosemide at high doses with HSS adds an important hydrosaline renal excretion because the advancement in renal blood flow allows the optimal concentration of furosemide in the Henle’s loop (7).

Further literature recommends that HSS decreased inflammatory markers, including interleukin-6 and tumor necrosis factor (16). High inflammatory cytokines levels are associated with a poor prognosis in CHF (17), and it is available that HSS administration improves outcomes, partly by an anti-inflammatory mechanism (18).

**Table 1:** Baseline characteristics and laboratory findings of the patients

		**Control Group**	**Intervention Group**	***P*** **-Value**
f	Male/Female	7/7	8/6	0.70^a^
PMH and PDH (No.)	CHF	5	3	0.72^a^
	DM	6	6	
	HTN	7	9	
	CKD	3	4	
	Cirrhosis	1	1	
	Malignancy	2	2	
	NSAIDs	3	10	
Habit	Smoking	5	5	
Age; year (Mean ± SD)		67.29 ± 12.67	63.00 ± 13.21	0.40^b^
Weight; kg (Mean ± SD)		81.74 ± 19.82	84.71 ± 21.11	0.70^b^
Serum Osmolality; mOsm/Kg (Mean ± SD)		284.89 ± 7.64	283.61 ± 7.98	0.67^c^
Urine Osmolality; mOsm/Kg (Mean ± SD)		329.79 ± 90.45	349.14 ± 78.58	0.18^b^
Serum Sodium; mEq/L (Mean ± SD)		134.93 ± 4.20	135.21 ± 4.00	0.91^b^
Serum Potassium; mEq/L (Mean ± SD)		4.43 ± 0.68	4.32 ± 0.61	0.66^c^
Urea; mg/dL (Mean ± SD)		101.32 ± 72.04	76.21 ± 52.28	0.30^c^
Serum Creatinine; mg/dL (Mean ± SD) Estimated GFR; mL/min (Mean ± SD)		2.36 ± 1.22 42.88 ± 37.25	1.68 ± 0.81 68.72 ± 67.21	0.19^b^ 0.27^b^
Urinary Cystatin C; mg/L (Mean ± SD)		2.08 ± 0.93	1.81 ± 0.84	0.43^c^
Urine Output; mL/24 h (Mean ± SD)		1450 ± 1100	1254 ± 321	0.48^b^
Blood Pressure; mmHg (Mean ± SD)				
Systolic		122.50 ± 24.94	124.29 ± 14.39	0.38^b^
Diastolic		72.86 ± 9.35	72.14 ± 8.02	0.80^b^
Heart Rate; bpm (Median; Minimum-Maximum)		82; 74-92	78.5; 72-110	0.23^b^
Uric Acid; mg/dL (Mean ± SD)		7.46 ± 2.77	6.82 ± 2.75	0.54^c^
Urinary Random Sodium; mEq/L (Mean ± SD)		93.91 ± 38.37	92.69 ± 26.07	0.92^c^
Serum Albumin; g/dL (Mean ± SD)		3.57 ± 0.47	3.48 ± 0.43	0.60^b^

^a^based on Chi-square test, ^b^based on Mann-Whitney U test, ^c^based on Independent Sample T-test. CHF: Congestive Heart Failure; DM: Diabetes Mellitus; HTN: Hypertension; CKD: Chronic Kidney Disease; NSAIDs: Non-Steroidal Anti Inflammatory Drugs.

**Table 2:** Characteristics and laboratory findings of the patients after the intervention (Day 3).

	**Control Group**	**Intervention Group**	***P*** **-Value**
Weight; Kg (Mean ± SD)	78.14 ± 19.04	78.33 ± 20.26	0.98^b^
Weight change; Kg (Mean ± SD)	3.59 ± 2.12	6.38 ± 2.17	0.002^b^
Serum Osmolality; mOsm/Kg (Mean ± SD)	291.16 ± 9.02	292.38 ± 3.57	0.64^b^
Urine Osmolality; mOsm/Kg (Mean ± SD)	358.21 ± 78.28	355.29 ± 102.76	0.84^a^
Serum Sodium; mEq/L (Mean ± SD)	137.79 ± 3.14	138.86 ± 2.35	0.10^a^
Serum Potassium; mEq/L (Mean ± SD)	3.98 ± 0.63	4.11 ± 0.53	0.33^a^
Urea; mg/dL (Mean ± SD)	113.75 ± 73.91	138.86 ± 2.35	0.31^a^
Serum Creatinine; mg/dL (Mean ± SD)	2.56 ± 1.38	1.74 ± 0.75	0.12^a^
Urinary Cystatin C; mg/L (Mean ± SD)	2.44 ± 0.90	2.04 ± 0.80	0.23^b^
Urine Output; mL/24 h (Mean ± SD)	2039 ± 682	2282 ± 790	0.39^b^
Blood Pressure; mmHg (Mean ± SD)			
Systolic	117.14 ± 17.94	114.64 ± 12.00	0.67^b^
Diastolic	69.29 ± 8.74	69.64 ± 6.93	0.77^a^
Heart Rate; bpm (Median; Minimum-Maximum)	80; 72-96	78.5; 70-110	0.77^a^
Uric Acid; mg/dL (Mean ± SD)	8.19 ± 2.78	6.85 ± 1.56	0.13^b^
Urinary Random Sodium; mEq/L (Mean ± SD)	101.86 ± 30.53	98.38 ± 28.10	0.95^b^

^a^based on Mann-Whitney U test. ^b^based on Independent Sample T-test.

## Limitation

The major limitation of the study was that we enrolled subjects with different fluid retention etiologies.

## Conclusion

Our findings suggest that the administration of HSS as an adjunct to loop diuretics may provide an effective treatment for increasing urine output and decreasing weight in patients with heart failure.
